# Deep learning to detect macular atrophy in wet age-related macular degeneration using optical coherence tomography

**DOI:** 10.1038/s41598-023-35414-y

**Published:** 2023-05-22

**Authors:** Wei Wei, Joshua Southern, Kexuan Zhu, Yefeng Li, Maria Francesca Cordeiro, Kirill Veselkov

**Affiliations:** 1 Department of Surgery and Cancer, Imperial College London, London, UK; 2 Ningbo Medical Center Lihuili Hospital, Ningbo, China; 3 Imperial College Ophthalmology Research Group, London, UK; 4 Computing, Imperial College London, London, UK; 5 School of Cyber Science and Engineering, Ningbo University of Technology, Ningbo, China

**Keywords:** Macular degeneration, Computational models

## Abstract

Here, we have developed a deep learning method to fully automatically detect and quantify six main clinically relevant atrophic features associated with macular atrophy (MA) using optical coherence tomography (OCT) analysis of patients with wet age-related macular degeneration (AMD). The development of MA in patients with AMD results in irreversible blindness, and there is currently no effective method of early diagnosis of this condition, despite the recent development of unique treatments. Using OCT dataset of a total of 2211 B-scans from 45 volumetric scans of 8 patients, a convolutional neural network using one-against-all strategy was trained to present all six atrophic features followed by a validation to evaluate the performance of the models. The model predictive performance has achieved a mean dice similarity coefficient score of 0.706 ± 0.039, a mean Precision score of 0.834 ± 0.048, and a mean Sensitivity score of 0.615 ± 0.051. These results show the unique potential of using artificially intelligence-aided methods for early detection and identification of the progression of MA in wet AMD, which can further support and assist clinical decisions.

## Introduction

Age-related macular degeneration (AMD) is the leading cause of visual impairment and irreversible blindness worldwide in the elderly population^1^. It is estimated to increase to 288 million in 2040^2^, approximately 8.7% of all global blindness^3^, due to a rapid increase in the aging population. AMD is a progressive degenerative disorder affecting the macula characterized by retinal drusen deposits, retinal pigment epithelium (RPE) abnormalities, and in the advanced stages, geographic atrophy of the RPE and the choriocapillaris, and choroidal neovascularisation (CNV). Although AMD may experience different stages (early, intermediate, late^4^), the endpoint of AMD is macular atrophy (MA), which is characterized by the permanent loss of photoreceptor cells, RPE, and underlying choriocapillaris^5^, either in advanced dry AMD (Geographic atrophy) or wet AMD (neovascular AMD)^6,7^, Furthermore, neovascularization and atrophy can coexist in the same eye^6^. Currently, no effective treatments are proven for MA, but tools for its early detection are an urgent and unmet need.

Historically, conventional methods to detect MA are based on color fundus photography (CFP) and fundus autofluorescence (FAF). Although both have played a significant part during the past two decades, they inevitably have their limitations. The biggest drawback of using CFP alone is the presence of an indistinct MA edge and difficult quantification of MA area^8^. The limitation of FAF is that the central macular luteal pigment absorbs the blue excitation light, making it impossible to determine foveal activity by using FAF images alone^9^. In contrast, optical coherence tomography (OCT) is an advanced technology which has fast become a leading and preferred tool to detect and assess MA in the retina^10–12^, because it is non-invasive, fast, and quantitative. OCT enables acquisition of volumetric images, including 25–61 scans and an en-face near infrared reflectance (NIR) image at the same time in a fast manner and provides a broad scale to evaluate MA in the retina.

In 2018, a new consensus to define MA using OCT was published by the Classification of Atrophy Meetings (CAM) group^8^. The classification is based on different stages of atrophy evolution, using four histological-OCT features: complete RPE and outer retinal atrophy (cRORA), incomplete RPE and outer retinal atrophy (iRORA), complete outer retinal atrophy (cORA), and incomplete outer retinal atrophy (iORA)^8^. The criteria to define cRORA have been suggested as follows: (1) a region of hypertransmission of at least 250 um in diameter, (2) a zone of attenuation or disruption of the RPE of at least 250 um in diameter, (3) evidence of overlying photoreceptor degeneration, and (4) absence of scrolled RPE or other signs of an RPE tear^8^. Meanwhile, when (1) the region of hypertransmission is less than 250 um in diameter, and (2) the zone of attenuation or disruption of the RPE is less than 250 um in diameter with or without the persistence of basal laminar deposits, it is defined as iRORA^8,13,14^. The findings of photoreceptor degeneration are often accompanied by disruption of the external limiting membrane (ELM), the ellipsoid zone (EZ), and the interdigitation zone (IZ)^13^. Therefore, it shows an absence of ELM, EZ and IZ zone in cRORA and iRORA, while it shows non-visibility of EZ and IZ zone with intact RPE in cORA and continuous disruption of EZ and IZ zone with intact RPE in iORA.

These standard criteria mentioned above enable a more accurate and detailed definition of MA allowing better monitoring of its progression using OCT. However, it depends currently on human graders, manual segmentation and subjective evaluation. This is time-consuming and labor-intensive because of the analysis of large volumetric scans, inter-grader variability and human bias^15^. Furthermore, it is non-scalable and unrealistic as standard procedure in real-world clinic practice.

Deep learning (DL) is being increasingly and intensively used to analyse ophthalmologic images because of its powerful ability to deal with bigdata objectively and efficiently^16,17^. The potential of DL for detecting early lesions and monitoring disease progression has been recognized, and it has become a leading analytical tool for retinal images, with the ability to detect structural changes objectively, stage pathological disease, and locate detailed lesions in the retina^18^. This computer-based technology can be used in image segmentation, automatic classification, data analysis, and quantification^19^. DL is widely applied to retinal layer segmentation and fluid segmentation^18^. In addition, DL models are also widely focused on lesion segmentation and classification of MA, but not on the progression of MA^20^. Further work is steadily advancing, focusing on abnormal structures associated with progression^20^. A promising application is to use DL algorithms for automated detection of MA from OCT scans. This would enable a reliable and reproducible method which can objectively detect lesions and avoid human bias and reader burden.

Several automated algorithms for measuring and quantifying areas of atrophy have been developed, with some attempts at using these to predict regions of MA growth, enlargement rate, and foveal involvement^21^. Niu et al.^22^ reported a fully automated algorithm that could predict the progression of MA growth in dry AMD using OCT segmentation and feature extraction. Zhang et al.^15^ developed a DL-OCT based model to identify the end stage of MA in dry AMD with a larger sample size and external validation. Liefers et al.^23^ extracted 13 most common features in the retina and developed a convolutional neural network (CNN) model for feature segmentation, including two atrophic featuresl. This model had only slightly higher sensitivity and accuracy than human graders^23^. Similarly, Derradji et al.^24^ developed a fully automated method (CNN) to detect and measure MA in dry AMD.

However, most studies have focused on MA in dry AMD^15,17,22,24–26^, and little work has been done to detect MA in wet AMD. Our study presents a fully automated algorithm to detect and quantify all six atrophic features of MA in wet AMD in OCT—namely: interrupted outer retina, interrupted RPE, absence of outer retina, absence of RPE, hypertransmission < 250um, and hypertransmission ≥ 250um.

## Results

### Data annotation

**Figure 1:**
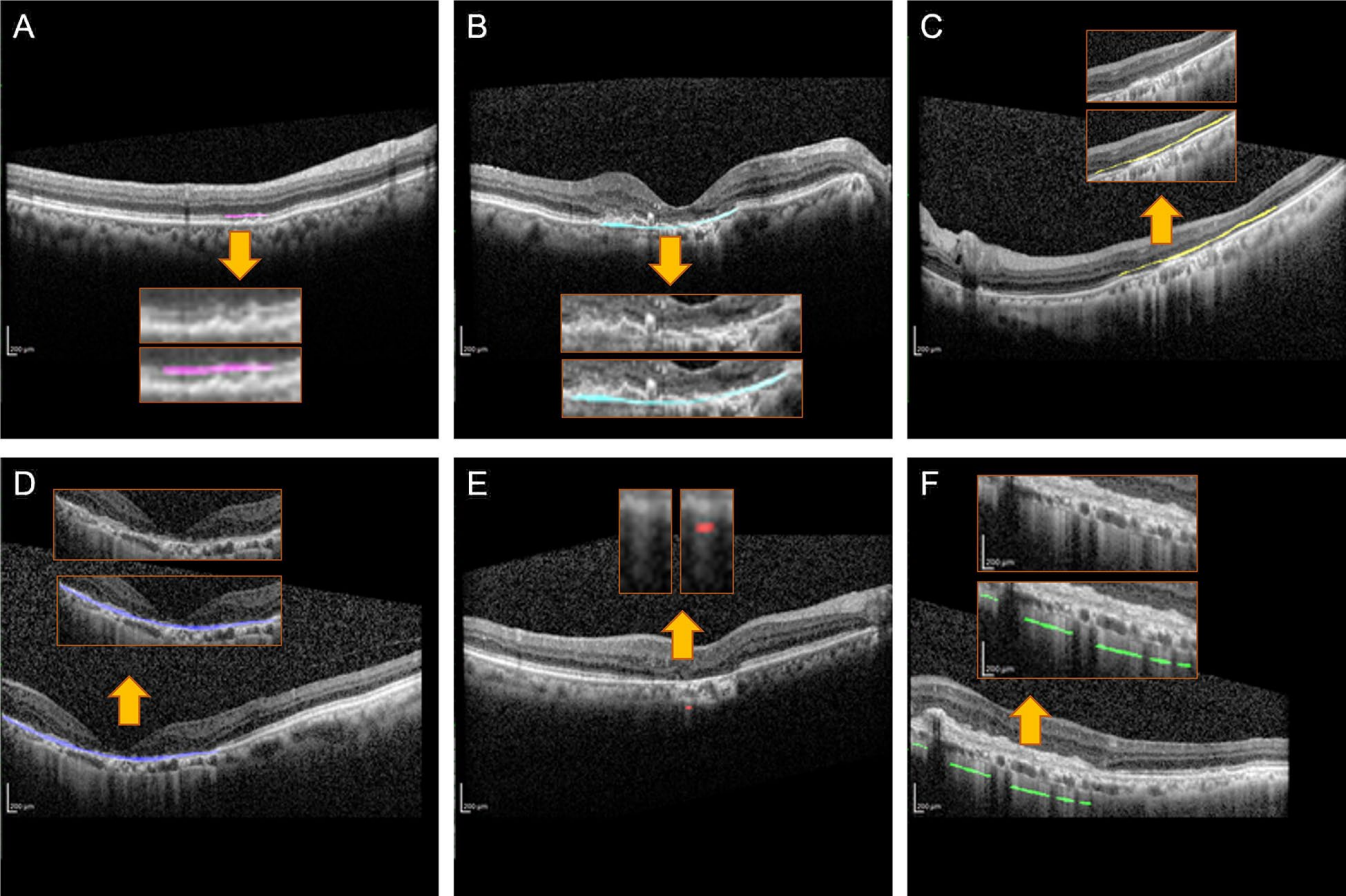
Representative examples of manual annotations. The six different features are demarcated as follows: (**A**) interrupted outer retina (pink); (**B**) interrupted RPE (lake blue); (**C**) absence of outer retina (yellow); (**D**) absence of RPE (dark blue); (**E**) hypertransmission < 250um (red); (**F**) hypertransmission ≥ 250um (green).

6503 manual annotations were performed using Labelbox (an open-source annotation software), and the distribution of annotated features is listed below (Table 1). The different lesions were manually annotated using different colors to distinguish them (Fig. 1.).

**Table 1:** Distribution of each manual annotation.

Classifications	Numbers of annotations	Pixel counts
Interrupted outer retina	704 (11%)	170,267 (0.018%)
Interrupted RPE	1477 (23%)	916,444 (0.095%)
Absence of outer retina	1208 (18%)	634,309 (0.075%)
Absence of RPE	439 (7%)	256,470 (0.026%)
Hypertransmission < 250um	1721 (26%)	37,783 (0.004%)
Hypertransmission ≥ 250um	954 (15%)	101,730 (0.010%)
Total	6503	969,562,332 (including background)

### Learning curve analysis

**Figure 2:**
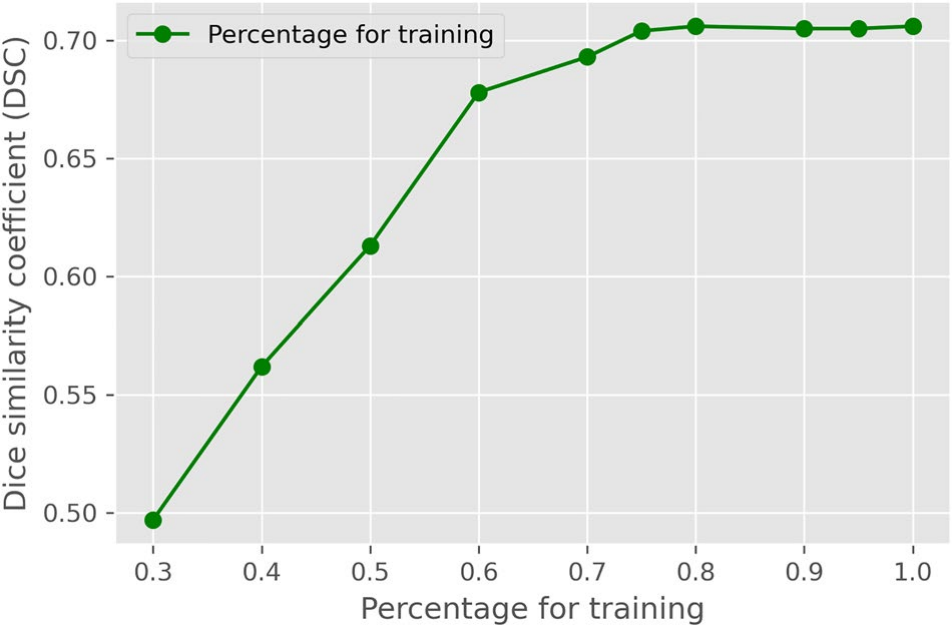
Performance on different sizes of training data. There are 2211 images in total, with 80% of images (1784) for training, 10% of images (221) reserved for validation, and the rest 10% of images (316) for testing. Among these, 1784 images were separated randomly based on different percentages for training. A plateau was reached at 0.706 with an 80% sample size.

As expected, the DSC performance increased with sample size, plateauing when the percentage of the training dataset reached 80% with a performance of 0.706, which means the sample size is sufficient (Fig. 2). However, it still needs further improvement for better performance.

### Automatic segmentation from the model

**Figure 3:**
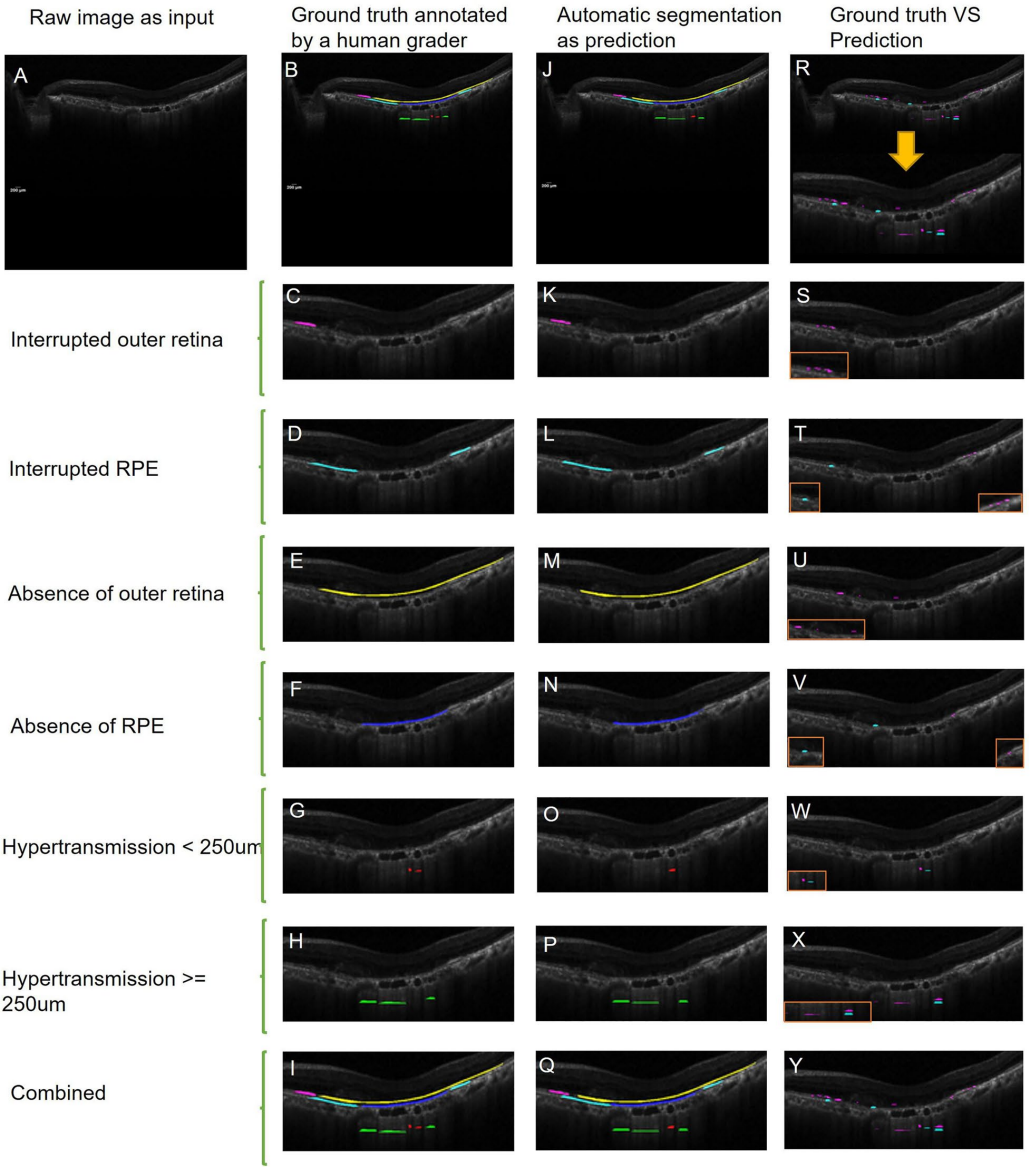
An example of prediction from models. A raw image (**A**) was fed into models as input. From left to right: name of each model, ground truth manually annotated by a human grader (**B**–**I**), automatic segmentation as prediction (**J**–**Q**), comparison between ground truth and prediction(**R**–**Y**) (magenta for true negative and cyan for false positive). The combined model can detect all six existing atrophic features associated with MA in wet AMD.

During independent testing, raw images were fed into the model that was already developed by training datasets, and automatic segmentation as the prediction was output through computational decision-making. Finally, the prediction was compared to manual annotation (ground truth) (Fig. 3).

### Evaluation of models

#### Dice similarity coefficient (DSC)

The DSC scores of each feature were as follows: interrupted outer retina model 0.662 ± 0.036; interrupted RPE model 0.711 ± 0.034; Absence of outer retina 0.671 ± 0.021; Absence of RPE model was 0.711 ± 0.029; Hypertransmission < 250 model 0.604 ± 0.052; Hypertransmission ≥ 250 model 0.640 ± 0.041. In comparison, the DSC score of the combined model was 0.706 ± 0.039, which is promising. From all these models, the DSC performance of the Hypertransmission < 250 and Hypertransmission ≥ 250 model was not as good as others because of the difficulty of the annotation itself.

#### Precision

The Precision scores of the six features were as follows: Interrupted outer retina model 0.861 ± 0.068; interrupted RPE model 0.848 ± 0.057; absence of outer retina model 0.839 ± 0.053; absence of RPE model 0.834 ± 0.047; hypertransmission < 250 model 0.786 ± 0.095; hypertransmission ≥ 250 model 0.793 ± 0.086. Finally, the Precision score of the combined model was 0.834 ± 0.048. The Precision metric of all the models obtained good performances overall.

#### Sensitivity

The Sensitivity scores of each of the six features were as follows: interrupted outer retina 0.540 ± 0.041; interrupted RPE 0.614 ± 0.040; absence of outer retina 0.560 ± 0.023; absence of RPE 0.619 ± 0.028; hypertransmission < 250 0.495 ± 0.051; hypertransmission ≥ 250 model 0.542 ± 0.047. The Sensitivity score of the combined model was 0.615 ± 0.051. The Sensitivity metric of all the models did not obtain high performances compared to the Precision metric.

#### Comparison of each model’s performance

**Figure 4:**
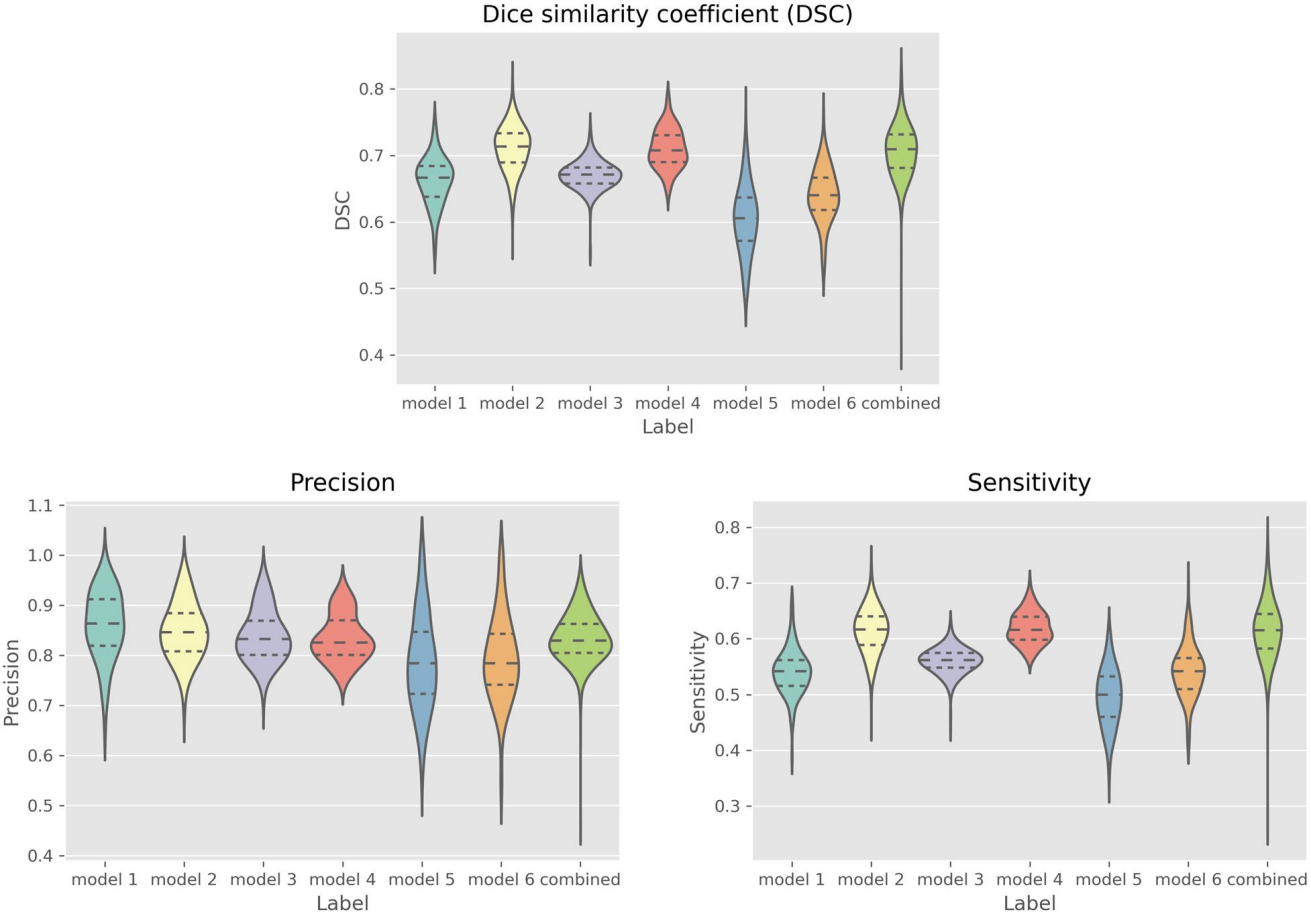
Comparison of each model’s performance. Model 1: interrupted outer retina; model2: interrupted RPE; model 3: absence of outer retina; model 4: absence of RPE; model 5: hypertransmission < 250um; model 6: hypertransmission ≥ 250um; combined: combined with all six atrophic features. Overall, the combined model got a promising performance in DSC, Precision, and Sensitivity. The DSC score of Hypertransmission, either in less than 250um or more than 250um, was slightly lower than others. The Precision score of each independent model was similar and relatively stable. However, the Sensitivity score of each independent model varied, and the performance of Sensitivity was moderately lower than the other two indicators on average.

A promising performance in DSC, Precision, and Sensitivity was obtained for the combined model. However, for each independent model, the results varied; the score of DSC and Precision was slightly higher than Sensitivity (Table 2, Fig. 4.). Overall, these fully automated CNN models were promising.

**Table 2:** A comparison of each model’s performance.

Classification	DSC	Precision	Sensitivity
Interrupted outer retina	0.662 ± 0.036	0.861 ± 0.068	0.540 ± 0.041
Interrupted RPE	0.711 ± 0.034	0.848 ± 0.057	0.614 ± 0.040
Absence of outer retina	0.671 ± 0.021	0.839 ± 0.053	0.560 ± 0.023
Absence of RPE	0.711 ± 0.029	0.834 ± 0.047	0.619 ± 0.028
Hypertransmission < 250um	0.604 ± 0.052	0.786 ± 0.095	0.495 ± 0.051
Hypertransmission ≥ 250um	0.640 ± 0.041	0.793 ± 0.086	0.542 ± 0.047
Combined	0.706 ± 0.039	0.834 ± 0.048	0.615 ± 0.051

## Discussion

MA is irreversible and significantly impairs visual acuity, but few clinical endpoints can be used to assess early treatment results and prediction thereof. There is still no widely applied treatment to prevent or delay MA progression until now; thus, early detection and regular monitoring of lesions in AMD patients is more crucial than ever before. The purpose of our study was to automatically identify and quantify MA at an early stage and predict the progression using accurate manual annotation as masks. Additionally, we aimed to highlight the progression features of MA. In this study, we developed a fully automated algorithm to detect all the atrophic features associated with MA in wet AMD, even at its early stage, which may provide individualized treatments in clinics and benefit both patients and clinicians.

MA progression is a gradually complicated process resulting in irreversible vision loss, and thus, early detection is essential for the development of novel therapis. Previous detection of MA was mainly based on CFP and FAF^6,27–29^. With the development of imaging technology, OCT has become a preferred imaging tool for assessment, especially combined with artificial intelligence (AI)^17,25,29^. This was highlighted by the CAM group who proposed new atrophy criteria on the basis of OCT imaging in 2018^8^. Different stages of atrophy are defined according to changes of retinal structure in different layers, that is, the presence or absence of the outer retina, RPE and hypertransmission. AI technology, especially using deep learning, can extract specific lesions that may be challenging or even invisible to the human eye^30^. Recently, Ronneberger, Olaf et al. have verified that layer segmentation analysis not only requires less computational capacity than other deep learning methods but also needs less dataset samples for training^31^. The Unet architecture is able to precisely detect various lesions as well as their localization^31^.Our model adopted this Unet architecture and it is a type of individual segmentation algorithm which is based on defined atrophic morphological changes in different retinal layers. The biggest strength of this strategy is it can monitor the overall progression of MA in the long term and provide a comprehensive visulization of individual lesions over time.

As far as we are aware, this is the first paper to automatically detect all six atrophic features of MA in wet AMD based on CAM criteria. It is relatively simple and easy to delineate atrophic features in dry AMD, but the atrophy in wet AMD is complicated because of the presence of multiple lesions with the same hyperreflective signals, like fluid, scar tissue, and subretinal hyperreflective materials. Despite this, we were able to obtain a good performance in all models, including DSC, Precision, and Sensitivity, and the overall DSC score was 0.706 in the combined model, the Precision score was 0.834 and Sensitivity score was 0.615. In addition, we compared our results to recent papers about automated detection of MA in AMD using OCT. These studies mainly focused on MA in dry AMD and only detected RORA, the end stage of MA, based on CAM consensus.

**Figure 5:**
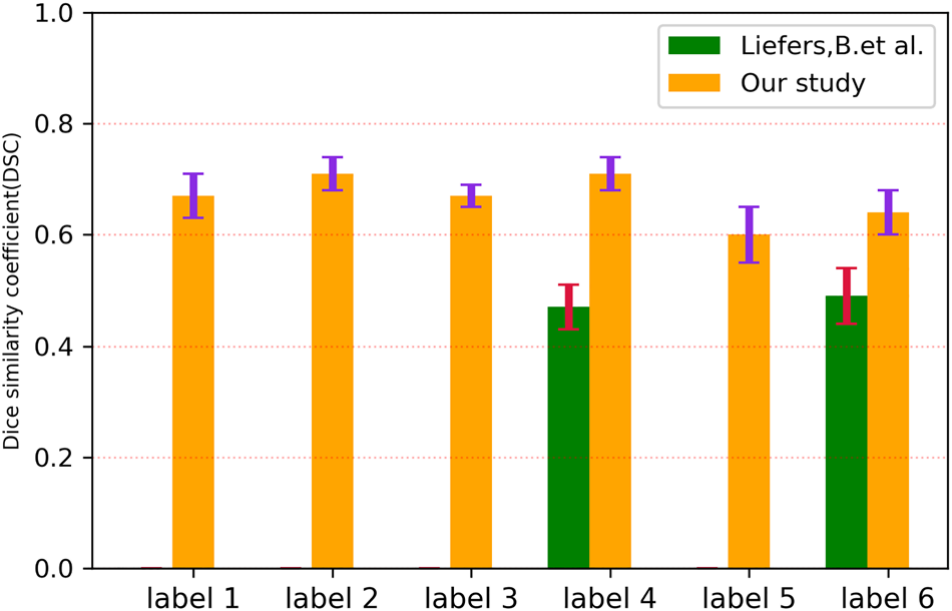
A comparison of OCT-AI based method to detect atrophic features in wet AMD. Label 1: interrupted outer retina; label 2: interrupted RPE; label 3: absence of outer retina; label 4: absence of RPE; label 5: hypertransmission < 250um; label 6: hypertransmission ≥ 250um. We detected six atrophic-associated features in wet AMD compared to Liefers, B’s study which was designed to detect only two atrophic-associated features (absence of RPE and hypertransmission ≥ 250um). When compared to these two features that were included in Liefers, B’s study, our performance is more promising.

Derradji et al.^24^ annotated the region of RORA in dry AMD as a rectangle using OCT and got more than 0.8 on average in DSC score. However, the annotation model was relatively simple and not designed to delineate small lesions accurately and quantitively without segmentation. Zhang et al.^15^ used a larger sample size to train a modified Unet model and even tried external validation. Although it was outstanding in both performance with internal and external validation (0.75–0.87 of DSC score), this model was based solely on RORA detection, the end stage of MA in dry AMD. Liefers et al.^23^ developed a model to detect 13 features in wet AMD, including two atrophic features including hypertransmission and RPE loss. However, these two features did not perform well, with only 0.47 and 0.49 DSC scores respectively^23^. Compared to these studies, we have detected all the relevant atrophic features of MA in wet AMD in our Unet architecture model and attained a promising performance overall. This was especially apparent in the performance of hypertransmission and absence of RPE (RPE loss) compared to the results of Liefers et al.^23^, which was also designed to detect some atrophic lesions in wet AMD (Fig. 5).

Although the overall Precision score was high, the performance of Sensitivity was relatively lower, which means high precision does not always mean high quality of Sensitivity (also called Recall)^32^. This suggests that it is hard to detect the atrophic lesions with high sensitivity and accuracy, attributable to the limitations of manual annotation, especially on the segmentation of hypertransmission. In support of this, it is important to note that the performance of hypertransmission was not as good as others as the manual annotation was based on the binary criteria of the width of hypertransmision, either less than 250 um or more than 250 um. Hence accurate localization of this lesion was not achieved. Maybe we could change the annotation manner next time, that is, we can segment the whole region of hypertransmission instead of the width, which is easier for the computer to learn the input information.

We have a smaller sample size than other studies^15,23,24^ because of the complicated situation in wet AMD, including poor image quality caused by cataracts and noncompliance with regular follow-up visits. To overcome the shortage of data, we annotated all the B-scans of OCT volumes of each patient, though several scans may not have enough atrophic features. On the contrary, some studies only chose scans that have distinct atrophic features^15,23^ in order to get sufficient atrophic features as labels, and their higher annotation partly ensures the superiority of the model because the computer can learn much input of features during the training stage. However, from our learning curve, it shows that the sample size is sufficient for training, mainly because there are sufficient annotated labels in our samples.

The study presented here also has several limitations. One of the limitations is the ability to generalize beyond external cohorts, which is always a common limitation of deep learning models. In our study, we did not give an external validation either in AMD patients or in the general population because of the difficulty of collecting long-term follow-up data in wet AMD with high resolution. Another limitation is that the automated model here only detects morphological changes of atrophy in OCT, not including other features that always occur in wet AMD, such as subretinal or intraretinal fluid, pigment epithelial detachment, and subretinal hyperreflective material. The reason is that too many annotations mean unavoidable overlappings, which may result in the low performance of models. Therefore, it is still a long way to develop a comprehensive model in wet AMD including all structural changes, and apply this comprehensive model in clinics as a screening tool for AMD progression. Other limitation is that our model trained, validated and tested is only based on Spectralis SD-OCT, a widely used OCT device; however, it has not been performed on other available OCT devices.

In summary, we have developed a promising fully automated model to detect all six main atrophic features associated with MA in wet AMD. Although this is from a relatively small sample size, we believe that further optimization of the automated CNN model will address outlined limitations described above and lead to a better-performing model with huge potential in retinal medical clinics all over the world. We believe that using our comprehensive automated analysis will enable the detection of MA at its earliest stages, allowing early intervention and increasing the time window of therapeutic opportunity—thereby preventing vision loss. In addition, this OCT-DL based algorithm can evaluate the effectiveness of drugs and monitor the progression of MA both for medical research and clinics. Ultimately, this should be a great advance in personalized patient management.

## Methods

### Ethics of clinical research

This retrospective and observational study was conducted by analyzing the electronic medical records of wet AMD patients treated at Ningbo medical center, Lihuili Hospital, China. The clinical study was conducted in accordance with the World Medical Assembly declaration of Helsinki and other relevant regulations.

Prior to the start of the study, the protocol was approved by the ethics committee in Ningbo medical center, Lihuili Hospital (KY2021PJ126). Before every subject was included in this study, the researchers had the responsibility to the participants to complete a comprehensive introduction to the purpose of this study and potential risks, followed by signing a written informed consent. The privacy of subjects and confidential data was protected throughout the study.

This study forms part of a larger study based on a large sample database where data-sharing has already been approved by Ningbo medical center, Lihuili Hospital, China.

### Patient population and data collection

This retrospective study included patients of wet AMD who showed MA at baseline and were followed up after their first anti-vascular endothelial growth factor (anti-VEGF) injection. The treatment included cases of stopping and switching to other anti-VEGF drugs between 2018 and 2020. Exclusion criteria consist of (1) severe systemic diseases (e. g. cardiovascular disease), (2) presence of retinal pigment epithelial tear, (3) previous ocular surgery except for routine cataract surgery, (4) severe ocular diseases (e. g. diabetic retinopathy, uncontrolled glaucoma), (5) poor imaging quality.

Each patient was assessed monthly by OCT. The OCT device used and settings remained constant throughout the visits. The OCT volumes were acquired using a Spectralis HRA + OCT device (Heidelberg Engineering, Heidelberg, Germany). Each OCT volumetric scan included 25–61 cross-sectional B-scans. All the images used in the analysis were totally anonymized. Finally, 45 volumetric scans from 8 patients were recorded, and a total of 2211 raw images were collected.

### Image processing

volumetric cross-sectional B-scans of wet AMD patients with MA were collected retrospectively by OCT during each follow-up. After being imported into ImageJ (open-source software for image processing and analysis in Java), all these JPEG files were manually cropped to the same pixels (770*706) with a standardized scalebar, and then exported to Labelbox (an open-source annotation software) in PNG format for further expert annotation.

### Data annotation

All these 2211 anonymized images were annotated by an experienced grader and further confirmed by another independent expert based on CAM criteria^8^ in Labelbox (an open-source annotation software). Detailed annotation labels included layer segmentation and abnormal structural changes: Interrupted outer retina, Interrupted RPE, Absence of outer retina, Absence of RPE, Hypertransmission < 250um, and Hypertransmission ≥ 250um. The definition of “interrupted” is discontinuous and incomplete layer, and the definition of “absence” is the complete loss of the layer. Each classification of labels had a unique color. After annotation, all these images with annotation labels were exported from Labelbox in PNG format with raw images, label masks, and combined masks, which were further used to develop a CNN model. We used Python scripts to extract each label mask based on its unique RBG values and generate regarding masks respectively.

### Data and model development

The model was trained on a Windows PC with a 12 Gb NVIDIA GTX 3080 graphics card. Seven different models were trained to predict class specific regions of interest using an Unet network architecture inspired by Ronneberger in 2015^31^. Images were resized from their original resolution to 256*256 pixels in order to be consistent with the input requirement of Unet architecture. Each model was trained for 300 epochs with a batch size of 16, and parameters were optimized using Adam optimizer proposed in 2015^33^ and the output threshold was 0.5. The 2211 annotated OCT images were randomly split into three datasets at patient level, training datasets (0.8, 1784), validating datasets (0.1, 221) and testing datasets (0.1, 316).

**Figure 6:**
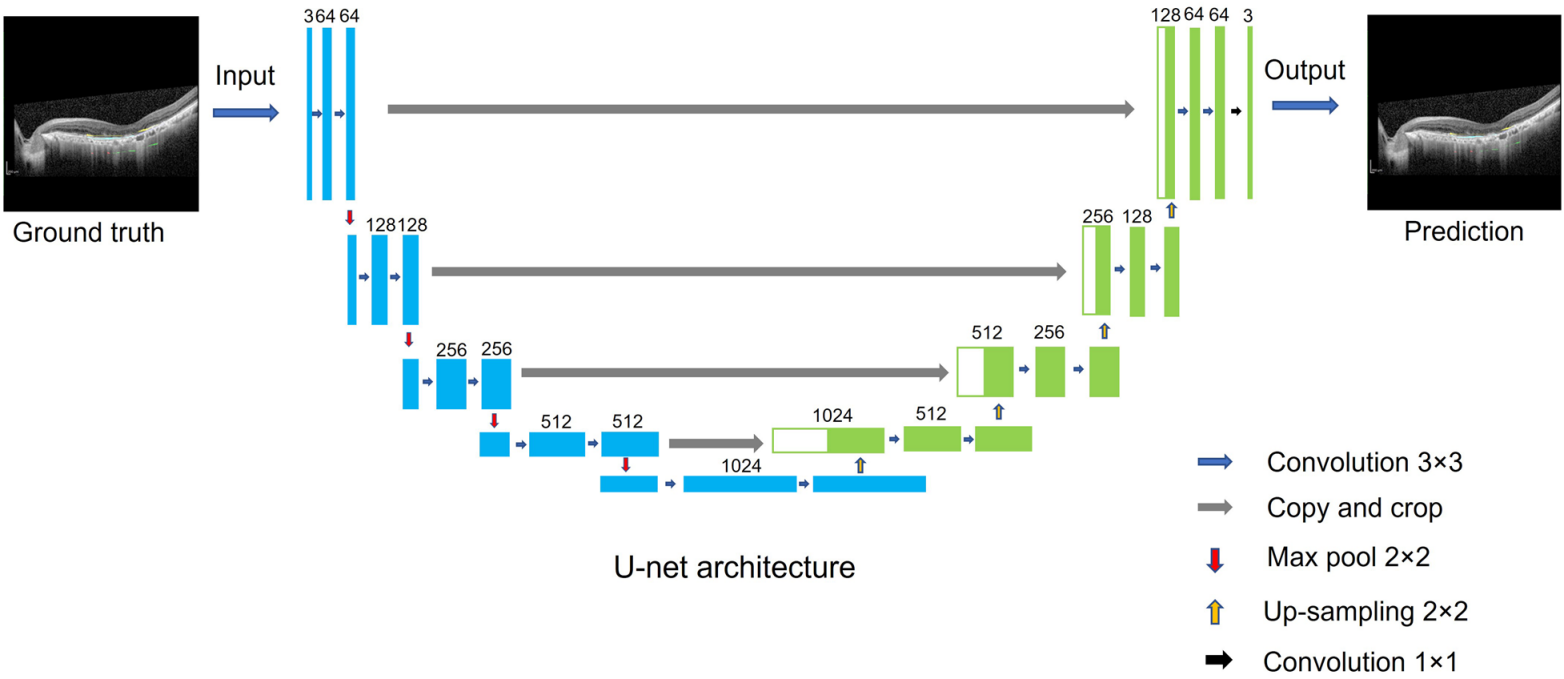
Introduction of U-net architecture. The raw images and label masks (ground truth) were input into the model for training. This architecture consists of a contracting section (left side) and an expanding section (right side). After convolution, max pool, and up-sampling, the model output prediction images as the outcome of computer learning.

U-net is a cutting-edge algorithm for semantic segmentation. It is based on fully convolutional networks using encoder-decoder network architecture^34^. The architecture was introduced by Olaf Ronneberger and his team in 2015^31^. The architecture of U-net looks like the shape of “U” which defines its name. This architecture consists of a contracting path to capture context and a symmetric expanding path that enables precise localization. Each path includes many blocks, such as the convolutional layer, max pooling, and up-sampling layer. We developed this U-net model using the Pytorch framework in a Python environment based on Anaconda software (Fig. 6).

We trained six U-net models separately first based on annotation labels: Interrupted outer retina model, Interrupted RPE model, Absence of outer retina model, Absence of RPE model, Hypertransmission < 250um model, and Hypertransmission ≥ 250um model. After that, we finally trained a combined automated model and ensured every pixel was uniquely classified into one of the six regions of interest without overlapping.

### Statistical analysis

Dice similarity coefficient (DSC), Precision, and Sensitivity were calculated to evaluate the models’ performance.

The DSC score was the primary outcome to evaluate the models. DSC is a spatial overlapping index for semantic segmentation, which is used to calculate the overlapping proportion of the ground truth and the prediction^35^. A DSC score ranges from 0 to 1, with 0 indicating no overlapping area and 1 indicating a fully overlapping area. The formula is as follows:

$${\text{DSC}} = \left( {{2}*{\text{Area}}\;{\text{of}}\;{\text{Overlapping}}} \right)/{\text{Total}}\;{\text{area}}\;{\text{covering}}\;{\text{annotated}}\;{\text{and}}\;{\text{predicted}}\;{\text{pixel}}\;{\text{regions}}$$

Precision, also called positive predictive value (PPV), shows the ability to predict true positives from all the positives^36^. The formula is as follows:

$${\text{Precision}} = {\text{TP}}/\left( {{\text{TP}} + {\text{FP}}} \right)\quad \left( {{\text{TP}}:{\text{True}}\;{\text{Positive}}\;{\text{FP}}:{\text{False}}\;{\text{Positive}}} \right)$$

Sensitivity, also known as Recall, or true positive value (TPV), shows the ability to detect true positive from all the prediction^36^. The formula is as follows:

$${\text{Sensitivity}} = {\text{TP}}/\left( {{\text{TP}} + {\text{FN}}} \right)\quad \left( {{\text{TP}}:{\text{True}}\;{\text{Positive}}\;{\text{FN}}:{\text{False}}\;{\text{Negative}}} \right)$$

### Learning curve analysis

To determine whether the sample size was sufficient to train the robust model, we performed a learning curve analysis. 1784 training images were randomly separated by setting different percentages (5%, 10%, 15%, etc.). Further DSC performance was evaluated on validating datasets to compare the DSC performance between each training model.

## Supplementary Information

Supplementary Information.

## Data Availability

The real-life clinical datasets used in current study are not publicly available due to privacy constraints. The data can be requested for sharing for peer-review or research purposes by contacting Wei Wei (w.wei20@imperial.ac.uk).

## Code availability

The Deep Learning models described in this work were developed using the Pytorch framework in a Python environment. The performance analysis was conducted using code developed in Python. The source code is fully opened on GitHub: https://github.com/ww1520/Macular-atrophy-AI-in-AMD.

## References

[1] Fleckenstein M (2021). Age-related macular degeneration. Nat. Rev. Dis. Primers.

[2] Wong WL (2014). Global prevalence of age-related macular degeneration and disease burden projection for 2020 and 2040: A systematic review and meta-analysis. Lancet Glob. Health.

[3] Ricci F (2020). Neovascular age-related macular degeneration: Therapeutic management and new-upcoming approaches. Int. J. Mol. Sci..

[4] Bird AC (1995). An international classification and grading system for age-related maculopathy and age-related macular degeneration. Surv. Ophthalmol..

[5] Fleckenstein M (2018). The progression of geographic atrophy secondary to age-related macular degeneration. Ophthalmology.

[6] Sadda SR, Tuomi LL, Ding B, Fung AE, Hopkins JJ (2018). Macular atrophy in the HARBOR study for neovascular age-related macular degeneration. Ophthalmology.

[7] Abdelfattah NS (2017). Macular atrophy in neovascular age-related macular degeneration with monthly versus treat-and-extend ranibizumab: Findings from the TREX-AMD trial. Ophthalmology.

[8] Sadda SR (2018). Consensus definition for atrophy associated with age-related macular degeneration on OCT: Classification of atrophy report 3. Ophthalmology.

[9] Lindner M (2015). Directional kinetics of geographic atrophy progression in age-related macular degeneration with foveal sparing. Ophthalmology.

[10] Giocanti-Auregan A (2015). Predictive value of outer retina en face OCT imaging for geographic atrophy progression. Invest. Ophthalmol. Vis. Sci..

[11] Schaal KB, Gregori G, Rosenfeld PJ (2017). En face optical coherence tomography imaging for the detection of nascent geographic atrophy. Am. J. Ophthalmol..

[12] Yehoshua Z (2011). Progression of geographic atrophy in age-related macular degeneration imaged with spectral domain optical coherence tomography. Ophthalmology.

[13] Guymer RH (2020). Incomplete retinal pigment epithelial and outer retinal atrophy in age-related macular degeneration: Classification of atrophy meeting report 4. Ophthalmology.

[14] Wu Z (2021). OCT signs of early atrophy in age-related macular degeneration: Interreader agreement: Classification of atrophy meetings report 6. Ophthalmol. Retina.

[15] Zhang G (2021). Clinically relevant deep learning for detection and quantification of geographic atrophy from optical coherence tomography: A model development and external validation study. Lancet Digit Health.

[16] Schlegl, T., Waldstein, S. M., Vogl, W.-D., Schmidt-Erfurth, U. & Langs, G. in *International Conference on Information Processing in Medical Imaging.* 437–448 (Springer). 10.1007/978-3-319-19992-4_34 26221693

[17] De Fauw J (2018). Clinically applicable deep learning for diagnosis and referral in retinal disease. Nat. Med..

[18] Sarhan MH (2020). Machine learning techniques for ophthalmic data processing: A review. IEEE J. Biomed. Health Inform..

[19] Lu W (2018). Applications of artificial intelligence in ophthalmology: General overview. J. Ophthalmol..

[20] Arslan J (2020). Artificial intelligence algorithms for analysis of geographic atrophy: A review and evaluation. Transl. Vis. Sci. Technol..

[21] Schmidt-Erfurth U, Klimscha S, Waldstein SM, Bogunović H (2017). A view of the current and future role of optical coherence tomography in the management of age-related macular degeneration. Eye (London).

[22] Niu S, de Sisternes L, Chen Q, Rubin DL, Leng T (2016). Fully automated prediction of geographic atrophy growth using quantitative spectral-domain optical coherence tomography biomarkers. Ophthalmology.

[23] Liefers B (2021). Quantification of Key retinal features in early and late age-related macular degeneration using deep learning. Am. J. Ophthalmol..

[24] Derradji Y (2021). Fully-automated atrophy segmentation in dry age-related macular degeneration in optical coherence tomography. Sci. Rep..

[25] Ji Z, Chen Q, Niu S, Leng T, Rubin DL (2018). Beyond retinal layers: A deep voting model for automated geographic atrophy segmentation in SD-OCT images. Transl. Vis. Sci. Technol..

[26] Xu R (2019). Automated geographic atrophy segmentation for SD-OCT images based on two-stage learning model. Comput. Biol. Med..

[27] Grunwald JE (2014). Risk of geographic atrophy in the comparison of age-related macular degeneration treatments trials. Ophthalmology.

[28] Kuehlewein L (2016). Predictors of macular atrophy detected by fundus autofluorescence in patients with neovascular age-related macular degeneration after long-term ranibizumab treatment. Ophthalmic Surg. Lasers Imaging Retina.

[29] Suri R, Neupane YR, Jain GK, Kohli K (2020). Recent theranostic paradigms for the management of age-related macular degeneration. Eur. J. Pharm. Sci..

[30] Schmidt-Erfurth U, Sadeghipour A, Gerendas BS, Waldstein SM, Bogunović H (2018). Artificial intelligence in retina. Prog. Retin Eye Res..

[31] Ronneberger, O., Fischer, P. & Brox, T. in *Medical Image Computing and Computer-Assisted Intervention–MICCAI 2015: 18th International Conference, Munich, Germany, October 5–9, 2015, Proceedings, Part III 18.* 234–241 (Springer).

[32] Su LT (1994). The relevance of recall and precision in user evaluation. J. Am. Soc. Inf. Sci..

[33] Kingma, D. P. & Ba, J. Adam: A Method for Stochastic Optimization. arXiv:1412.6980 (2014).

[34] Long, J., Shelhamer, E. & Darrell, T. in *Proceedings of the IEEE Conference on Computer Vision and Pattern Recognition.* 3431–3440.

[35] Zou KH (2004). Statistical validation of image segmentation quality based on a spatial overlap index. Acad. Radiol..

[36] Buckland M, Gey F (1994). The relationship between recall and precision. J. Am. Soc. Inf. Sci..

