# Supplementary material for: Deep learning to detect macular atrophy in wet age-related macular degeneration using optical coherence tomography
Source: Sci Rep. 2023 May 22;13:8296. doi: 10.1038/s41598-023-35414-y (PMC10203346; doi:10.1038/s41598-023-35414-y)
Supplement: Supplementary file 1 — Supplementary Information. [file 41598_2023_35414_MOESM1_ESM.docx]

**Supplementary Figure 1**


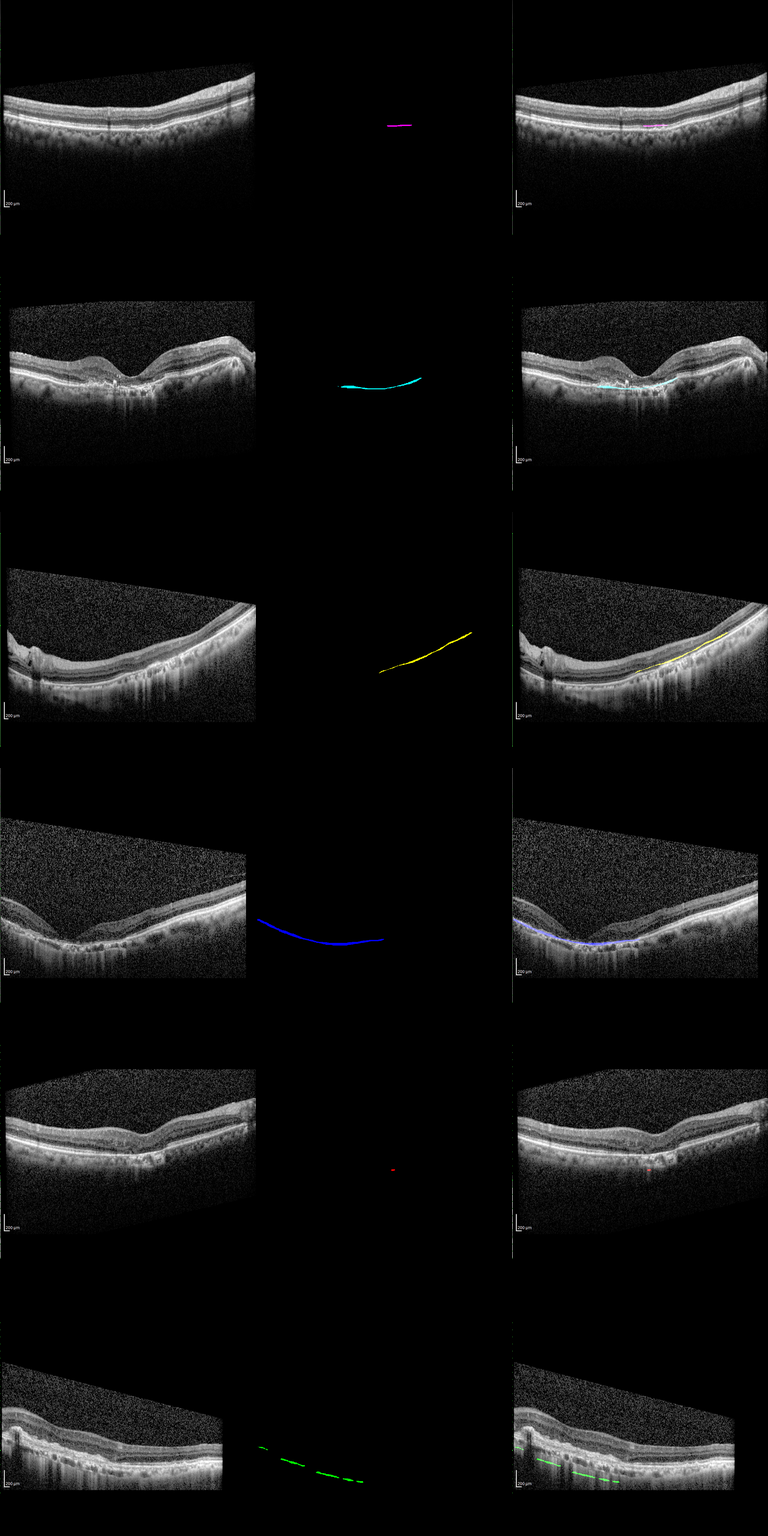


Figure S1. Representative examples of manual annotations. The left column shows raw OCT images (A-F), the middle column shows label masks (G-L) and the right column shows combined masks (M-R), The 6 different features are demarcated as follows: interrupted outer retina (pink); interrupted RPE (lake blue); absence of outer retina (yellow); absence of RPE (dark blue); hypertransmission < 250um (red) ; hypertransmission >=250um (green).

**Supplementary Table 1**

| **Authors** | **Year of publication** | **Sample size** | **Purpose of model** | **Method** | **Model network** | **Dice similarity coefficient (DSC)** |
| --- | --- | --- | --- | --- | --- | --- |
| Derradji, Y. et al.^1^ | 2021 | 2301 slides for training, 256 for validation and 1038 slides for a separate test | a fully automated method to detect and measure RORA in dry AMD | Annotated RORA as a rectangle in dry AMD | CNN | 0.881(grader 1 as ground truth)  0.844(grader 2 as ground truth) |
| Zhang, G. et al. ^2^ | 2021 | 3024 slides for training, 958 slides for tuning, and 1067 slides for validation | a fully automated method to detect and quantify MA in dry AMD | Annotated 3 atrophic features (RPE loss, overlying photoreceptor degeneration, and hypertransmission) in dry AMD | Modified U-Net | RPE loss: 0.76  Overlying photoreceptor degeneration: 0.87  Hypertransmission: 0.86  MA in dry AMD: 0.75 |
| Liefers, B. et al. ^3^ | 2021 | 2712 slides for training and 112 slides for validation | An automated method for segmentation of 13 features associated with wet AMD and atrophic AMD | Annotated 13 features in wet AMD (including 2 atrophic features: hypertransmission and RPE loss) | CNN | Hypertransmission :0.49,  RPE loss:0.47  Combined model :0.63 |
| Ours |  | 1784 slides for training, 221slides for validation and 316 slides for testing | An automated method for segmentation of all six atrophic features in wet AMD | Annotated all six atrophic features in wet AMD | U-Net | Interrupted outer retina: 0.662  Interrupted RPE: 0.711  Absence of outer retina: 0.671  Absence of RPE: 0.711  Hypertransmission <250: 0.604  Hypertransmission >=250: 0.640  Combined model: 0.706 |

Table S1. comparison of OCT-AI based methods to detect atrophic features in AMD

References

1 Derradji, Y. *et al.* Fully-automated atrophy segmentation in dry age-related macular degeneration in optical coherence tomography. *Sci Rep* **11**, 21893, doi:10.1038/s41598-021-01227-0 (2021).

2 Zhang, G. *et al.* Clinically relevant deep learning for detection and quantification of geographic atrophy from optical coherence tomography: a model development and external validation study. *Lancet Digit Health* **3**, e665-e675, doi:10.1016/s2589-7500(21)00134-5 (2021).

3 Liefers, B. *et al.* Quantification of Key Retinal Features in Early and Late Age-Related Macular Degeneration Using Deep Learning. *Am J Ophthalmol* **226**, 1-12, doi:10.1016/j.ajo.2020.12.034 (2021).
